# Whole Grains, Legumes, and Health

**DOI:** 10.1155/2012/903767

**Published:** 2012-03-29

**Authors:** Bernard Venn, Frank Thies, Carol O'Neil

**Affiliations:** ^1^Department of Human Nutrition, University of Otago, P.O. Box 56, Dunedin 9054, New Zealand; ^2^Section of Translational Medical Sciences, Division of Applied Medicine, School of Medicine and Dentistry, University of Aberdeen, Aberdeen AB243FX, UK; ^3^School of Human Ecology, Louisiana State University AgCenter, Baton Rouge, LA 70803, USA

The consumption of whole grains and legumes is recommended by public health agencies around the world. The recommendation to consume legumes is based on them being a good source of protein, fiber, and several micronutrients including iron and zinc. However, legumes tend to be consumed infrequently by many people in industrialized countries. Dietary interventions with legumes have yielded mixed results on health outcomes, and there may be reluctance by some people to increase the amount of legumes in their diets due to unfamiliarity with the food.

The recommendations for whole grains are based on purported health benefits of consuming whole grain over refined grain products. In comparison with their refined counterparts, whole grain foods tend to be higher in phytochemicals and fiber, and in several micronutrients including some of the B vitamins, magnesium, and selenium. Based on subjective dietary intake data, observational studies are reasonably consistent in their findings that higher cereal fiber and magnesium intakes are associated with lower risk of type 2 diabetes and cardiovascular disease. An objective marker of whole-grain intake would be useful, and in this regard, alkylresorcinols, compounds present in the bran particularly of wheat and rye, can be measured in biological fluids as potential biomarkers of wheat and rye intake.

Despite the recommendations, interventions with whole-grain foods have produced mixed results on health markers. One factor contributing to the variability in outcomes may be grain structure. Intact grain structure distinguishes “whole grain” from “whole meal,” a process in which the whole grains have been ground. The starch contained in an entirely or partially intact grain is surrounded by the fibrous seed coat. Chewing helps to release the starch from within the seed coat; however, not all of the starch will be released, and access by digestive enzymes will be hindered. This hindrance is often regarded as a good quality as it slows starch digestion and glucose absorption, aiding blood glucose control. Another factor in starch digestion is the type of fiber. Some grain fibers are water soluble and highly fermentable, and form viscous gels. The formation of the gels also slows down starch digestion and glucose absorption. A third factor associated with improved blood glucose control is the “subsequent meal effect,” a phenomenon in which the benefit of slowly absorbed carbohydrate on blood glucose at one meal is extended to the meal following. Thus, the recommendations to consume whole grain foods are supported by mechanistic plausibility, whilst the variability in outcomes of whole-grain and legume intervention studies may be related to differences in study design and compliance.

This special issue of the Journal of Nutrition and Metabolism explores some of the issues regarding the consumption of whole grains and legumes on health outcomes. It comprises three review and two research articles. Included is a comprehensive review of studies describing the “subsequent meal effect” in which mechanisms for the effect are discussed and research challenges suggested. The status of alkylresorcinols as biomarkers of whole-grain intake is presented that includes suggestions for further validation work and recommendations on how this might be achieved. The health benefits of beta-glucan, a fiber found in oat and barley bran, are reviewed including discussion of its effect when consumed as a component of the whole grain or as an extracted product. Mechanisms of action and challenges in the use of beta-glucans are discussed. The effect on postprandial blood glucose of incorporating whole grains into bread is reported with comparisons made among sprouted-grain, sourdough, and mixed grain breads in a group of overweight and obese men. At the other end of the nutritional spectrum, the effects of using locally grown grains and legumes as a porridge base with which to feed undernourished children are presented.

 General recommendations to consume whole-grain and legume foods appear to be well founded although there is much work to be done to identify grain/legume types, components, and intakes consistent with well-being among people of varying demographics and states of health. We hope that topics raised in this special issue lead to further research aimed at advancing our knowledge pertaining to whole-grain and legume consumption leading to strong evidence-based intake recommendations.

*Bernard Venn* *Bernard Venn* *Frank Thies* *Frank Thies* *Carol O'Niel* *Carol O'Niel*

